# *Helicobacter pylori* incidence and re-infection in the Aklavik *H. pylori* Project

**DOI:** 10.3402/ijch.v72i0.21594

**Published:** 2013-08-05

**Authors:** Sally Carraher, Hsiu-Ju Chang, Rachel Munday, Karen J. Goodman, the CANHelp Working Group

**Affiliations:** 1 Department of Anthropology, McMaster University, Hamilton, Canada; 2 Department of Medicine, University of Alberta, Edmonton, Canada; 3 Susie Husky Health Centre, Aklavik, Canada; 4 CAN*Help* Working Group, Division of Gastroenterology, University of Alberta, Edmonton, Canada

**Keywords:** Helicobacter pylori infection, Aboriginal health, collaborative research, epidemiology, incidence, Arctic

## Abstract

**Background:**

The Aklavik *H. pylori* Project (AHPP) (www.canhelpworkinggroup.ca) is a community-driven project examining *Helicobacter pylori* infection and its influence on health in a diverse Aboriginal community in the Northwest Territories. Initial research revealed that 58% of 333 participants who underwent a urea breath test (UBT) between 2007 and 2010 were *H. pylori*-positive. From 2008 to 2010, we offered treatment to *H. pylori*-positive participants and 113 consented to this treatment.

**Objective:**

We estimated *H. pylori* incidence in AHPP participants who initially tested negative and the re-infection frequency in initially positive participants who were successfully treated to clear the infection.

**Methods:**

Participants who were initially *H. pylori-*negative or negative after treatment during 2008–2010 were eligible for inclusion. From November 2011 to June 2012, participants were offered a UBT and the samples were analyzed using infrared spectroscopy (IRIS). Participants with a positive test result were classified as new cases for estimating incidence among participants testing negative at baseline and re-infection among those successfully treated for *H. pylori* infection.

**Results:**

Among 38 initially negative participants, follow-up UBT showed that 33 remained negative, 3 were positive, and 2 had uncertain status. The estimated incidence proportion during the follow-up period was 8.3% (95% CI: 1.8–22.0%). Among 43 participants with a negative post-treatment UBT, 41 remained negative and 2 were positive. The estimated re-infection proportion during the follow-up period was 4.7% (95% CI: 0.6–16.0%). The frequency of new cases was similar in males and females. Aboriginal participants had a combined re-infection/incidence rate of 2.4% per year (95% CI: 0.8–5.9% per year). All 9 non-Aboriginal participants remained free from infection throughout the study period, as did all 23 participants aged 55 years and above.

**Conclusions:**

The AHPP has substantially reduced the burden of infection in Aklavik since 2008. Continued monitoring, treatment, community engagement and knowledge translation activities are needed to ensure a lasting benefit of the project.

The Aklavik *H. pylori* Project (AHPP) is a community-driven project examining *Helicobacter pylori* infection and its influence on health in a diverse Aboriginal hamlet in the Northwest Territories, conducted by the Canadian North *Helicobacter pylori* (CAN*Help*) Working Group (http://www.canhelpworkinggroup.ca). The CAN*Help* Working Group brings together health care providers, Aboriginal government representatives, the Aklavik Health Committee (AHC), and University of Alberta researchers to address community concerns regarding the high prevalence of *H. pylori* infection in Aklavik and the association of this infection with stomach cancer.

Aklavik is an Arctic community of 633 people (according to the 2011 census) including predominately Inuvialuit and Gwich'in peoples, along with smaller numbers of Métis, other First Nations and non-Aboriginal residents. Overall project goals include investigating the impact of *H. pylori* infection on Canadian Arctic communities; identifying effective treatment strategies; developing recommendations aimed at *H. pylori* infection management; and effectively communicating research findings to address community concerns (1,2). Currently, there is no standard recommendation for community surveillance of *H. pylori* infection or, more generally, for testing and treating people who are not seeking medical care for relevant symptoms. However, the Maastricht IV/Florence Consensus Report (3) on clinical management of *H. pylori* infection recommends that, “A screen-and-treat strategy of *H. pylori* should be explored in communities with a significant burden of gastric cancer.” With guidance from the locally organized AHC, the AHPP developed approaches to participant recruitment, enrolment, *H. pylori* screening, and treatment in order to provide answers to community questions about the infection and its potential health consequences. During initial project planning, the AHC wanted all residents of Aklavik to be invited to undergo screening for *H. pylori* infection and treatment to be made available to all participants testing positive. A treatment trial conducted from 2008 to 2010 was designed to accommodate this request as well as to find effective treatments for the strains of *H. pylori* that exist in Aklavik and generate information that may be useful to regional health authorities in future policy development.

Initial research revealed that of the 333 project participants who were screened between November 2007 and June 2010, 193 (58%) were *H. pylori*-positive by urea breath test (UBT). From 2008 to 2010, the project offered treatment to *H. pylori*-positive participants. The aim of this new project component is to estimate the *H. pylori* incidence and re-infection rates in Aklavik since the treatment component of the AHPP was conducted.

## Background

*H. pylori* are gram-negative spiral-shaped bacteria uniquely adapted to live in the stomach lining of humans. *H. pylori* infection is often chronic and usually established during childhood. While a majority of those with chronic infection remain asymptomatic and free of disease consequences, it has been estimated that chronic *H. pylori* infection is responsible for more than 90% of peptic ulcer disease cases in developing countries where infection rates are high, and 65% of all gastric cancers worldwide (4,5). While the bacterium's pathogenic role in initiating gastritis, peptic ulcers and gastric cancers was described soon after *H. pylori* was identified in the late 20th century, *H. pylori* research in the public health sector has developed much more slowly. As the exact transmission pathway(s) remain unconfirmed, much current research aims to identify potential risk factors for specific populations and communities and to investigate the success and cost-effectiveness of particular surveillance and treatment programs.

Epidemiologic studies across the globe present a strong association of conditions, such as low socio-economic status (SES), low levels of education and marginalized ethnic status with *H. pylori* infection frequency (6). Particularly in early life, poverty and related issues including household crowding are commonly associated with higher *H. pylori* prevalence (7,8). Relatively high prevalence of *H. pylori* infection today exists in southeast Asia, Africa, Central and South America, and throughout the Arctic. Over the past 10 years, we have learned that *H. pylori* infection and gastric cancers are much more frequent in the Arctic regions of the United States, Canada, Scandinavia and Russia compared to the southern, more urban regions of these nations; and in Greenland compared to Denmark (9,10).

Research in the AHPP has revealed that similar social issues including income, ethnic status, parent education level and household crowding are associated with *H. pylori* infection (11). While we know that *H. pylori* is highly prevalent in several remote Arctic communities such as Aklavik, we know very little about *H. pylori* incidence and re-infection rates in the North. A systematic search reveals just one report of *H. pylori* incidence in northern Canada – from a follow-up study of Aboriginal children in northern Manitoba (12) – and no reports on re-infection. As a large proportion of Aklavik's community participated in screening and treatment for *H. pylori*, we were able to design an incidence and re-infection study, having identified participants who were negative for infection at the start of the study period. Our aim in this analysis is to estimate the incidence of new *H. pylori* infections in AHPP participants who initially tested negative (baseline-negative) and the re-infection frequency in initially positive participants who were successfully treated during the project (*H. pylori*-cleared).

## Methods

In 2008, the AHPP offered UBT screening to all Aklavik residents without restriction. During 2008–2010, 333 participants received a UBT to assess their infection status. The ages of the 333 project participants ranged from infancy to 80 years; 54% were female and 87% of them were Aboriginals (56% Inuvialuit, 27% Gwich'in and 4% other Aboriginal groups). A previous analysis compared demographic characteristics of project participants and the community population (13). The characteristics of the community population of Aklavik were estimated by the community profile of 2006 census. The findings suggested that the study population was older and comprised a slightly greater proportion of females than the community population.

From 2008 to 2010, the AHPP offered treatment to *H. pylori*-positive participants and 113 consented to treatment. The details and results of the trial have been presented elsewhere (14). Participants were randomized to receive either the standard triple therapy used across Canada (rabeprazole 20 mg po bid, amoxicillin 1 g po bid and clarithromycin 500 mg po bid for 10 days) or a sequential therapy (10 days of rabeprazole 20 mg po bid, days 1–5 amoxicillin 1 g po bid, days 6–10 clarithromycin 500 mg po bid and metronidazole 500 mg po bid), unless antibiotic susceptibility testing showed evidence of resistance to clarithromycin. For clarithromycin-resistant cases, individuals were randomized to receive either the sequential therapy or a quadruple therapy (10 days of bisthmuth 2 tablets qid, tetracycline 500 mg po qid, metronidazole 500 mg po tid and rabeprazole 20 mg po bid). Treatment success was assessed with a post-treatment UBT at least 10 weeks after the completion of treatment. Participants with treatment failures were treated again with a different regimen and reassessed at least 10 weeks after the completion of treatment.

For the new AHPP component aimed at estimating *H. pylori* incidence and re-infection rates, participants aged 15 years or above who were previously shown to be free of *H. pylori* infection were eligible for inclusion. All AHPP participants (n=179) who initially tested *H. pylori*-negative (n=107) or were negative after treatment (n=72) as of October 31, 2011 were invited to undergo a repeated UBT to classify their current *H. pylori* status. From November 2011 to June 2012, we offered participants UBT screening for *H. pylori* infection at the Susie Husky Health Centre in Aklavik. Breath samples were analyzed using infrared spectroscopy (IRIS) at the University of Alberta. We classified UBT results as positive (delta-over-baseline test values =4), negative (delta-over-baseline test values from −2 to 2.5) or borderline (test values between 2.5 and 4). For borderline results or breath samples with insufficient CO_2_ concentration, participants were asked to repeat the test. If a sample with a sufficient CO_2_ concentration could not be obtained, the result was considered unclassifiable and the participant was excluded from the analysis. When a repeat result was borderline, the borderline classification was retained. Participants with a positive test result were classified as new cases: incident cases in the baseline-negative group and re-infection cases in the *H. pylori*-cleared group.

We estimated two incidence measures: (a) the incidence proportion (the number of new cases divided by the number of infection-free participants who were retested for the incidence study) pertaining to the time between the negative UBT and the subsequent UBT and; (b) the incidence rate (the number of new cases divided by the person-time at risk). For estimating rates of incidence and re-infection events, we define the person-time at risk as the time period between the date of the last negative test and the date of the follow-up test for each person, subtracting half of this interval for participants with new infection onsets. We calculated the incidence rates by dividing the number of positive cases by the sum of person-time at risk.

Given its community-based research design, the AHPP is guided by community priorities. Previous analysis has shown that *H. pylori* infection is less frequent among non-Aboriginal community residents, and some community members requested that we examine whether there is a difference in infection frequency between the various Aboriginal groups residing in Aklavik (classified as “Inuvialuit,” “Gwich'in” and “other Aboriginal”). We report estimated incidence and re-infection frequencies with 95% confidence intervals (CI) for the total study population (n=79) and for demographic subgroups by age, sex and self-identified ethnicity. To compare incidence across groups, we estimated incidence rate differences and 95% CI. As individual follow-up periods vary, we do not compare incidence proportions in our analysis.

## Results

**Fig. 1:**
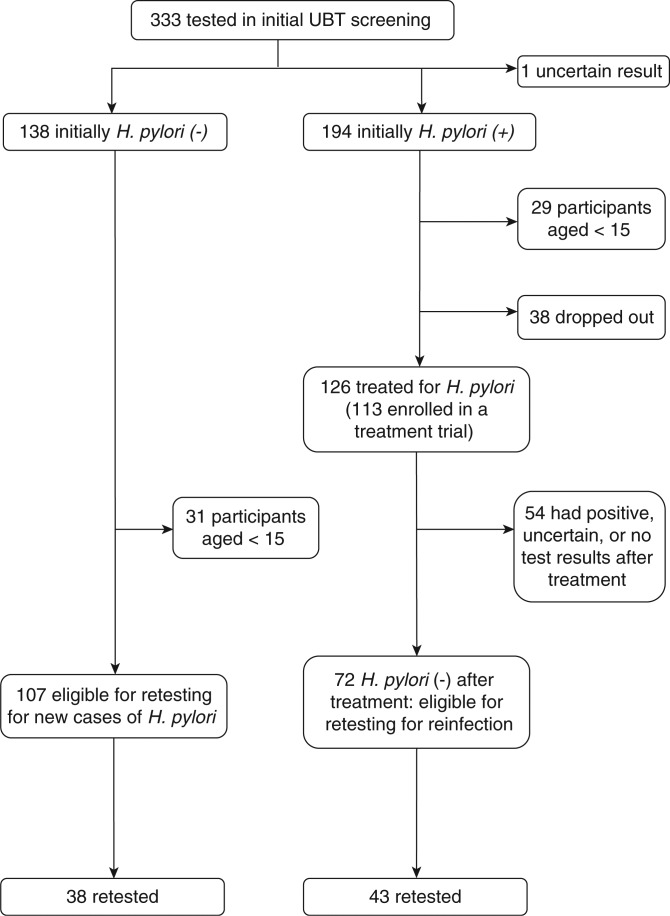
Participation of the incidence/re-infection study among participants aged≥15 years.

We retested 81 participants for this study (Fig. 1). Of these 81, age at the entry into this study period ranged from 18 to 80 years (mean: 43, median: 44); 45 were female (56%) and 66 self-identified as Aboriginal (81%). Among the 38 participants who had tested negative on initial screening, 33 remained negative on the follow-up UBT, 3 were positive and 2 had uncertain results. In this group, the estimated incidence proportion during the follow-up period was 8.3% (95% CI: 1.8–22%). Among the 43 participants with a previous negative post-treatment UBT, 41 remained negative and 2 were positive. The estimated re-infection proportion during the follow-up period was 4.7% (95% CI: −0.6–16.0%). We report person-years and mean and median person years-at-risk in Table I. As individual follow-up periods vary, we report both incidence proportions and rates, but only compared rates in our analysis.

**Table I:** Frequency of new *H. pylori* infections among participants in the Aklavik *H. pylori* Project, Northwest Territories, Canada

					Incidence	
					
	Tested, N	New cases	Person-years at risk	Mean (median)-years at risk	Proportion (%) 95% CI	Rate (% per year) 95% CI
Total	79*	5	239.3	3.0 (3.0)	(6.3) 2.1–14	(2.1) 0.7–4.9
Baseline-negative	36*	3	126.4	3.5 (3.8)	(8.3) 1.8–22	(2.4) 0.5–6.9
*H. pylori*-cleared	43	2	112.9	2.6 (2.8)	(4.7) 0.6–16	(1.8) 0.2–6.4
Male	32	1	94.8	3.0 (3.0)	(3.1) 0.1–16	(1.1) 0.3–4.7
Female	47	4	144.6	3.1 (2.8)	(8.5) 2.4–20	(2.7) 0.03–5.9
Age: 15–34 years	15	3	41.1	2.7 (2.8)	(20) 4.3–48	(7.3) 1.5–21.0
Age: 35–54 years	41	2	124.7	3.0 (3.0)	(4.9) 0.6–17	(1.6) 0.2–5.8
Age: 55 years and above	23	0	73.5	3.2 (3.0)	(0) 0–15†	(0) 0–5.0†
Inuvialuit‡	41	4	121.5	3.0 (2.9)	(9.8) 2.7–33	(3.3) 0.9–8.4
Gwich'in‡	23	1	68.3	3.0 (2.8)	(4.3) 0.1–22	(1.5) 0.04–8.2
Other Aboriginal‡	2	0	7.6	3.5 (3.8)	(0) 0–84†	(0) 0–49.0†
Non-Aboriginal‡	9	0	28.6	3.2 (3.2)	(0) 0–34†	(0) 0–13.0†

* Excludes 2 with uncertain results.

† One-sided, 97.5% confidence interval.

‡ Of 79 with classifiable results, excludes number of patients who declined to identify ethnicity.

Among the participants with follow-up results (n=79), the estimated rate of new cases was 2.1% per year (Table I) over an average follow-up of 3 years. Among people with a negative UBT at study entry, the estimated incidence rate was 1.8% per year; among people who had previously cleared an infection, the estimated re-infection rate was 2.4% per year. The rates in the 2 groups are similar, with the 95% CI for the rate difference of −0.6% per year comparing re-infection to new cases in those who were infection-free at baseline ranging from −4.2 to 3.0% per year.

### Results across groups: sex, age, ethnicity

Among participants with classifiable follow-up results, incidence proportions and rates (combining new cases of infection in participants who were *H. pylori*-negative at baseline and those who were successfully treated) were estimated for categories of sex, age and self-reported ethnic status. The estimated incidence proportion for the follow-up period was 3.1% (95% CI: 0.1–16.0%) in males and 8.5% (95% CI: 2.4–20%) in females. The estimated incidence rate was 1.1% per year in males and 2.8% per year in females. The estimated incidence rate difference comparing males to females was −1.7% per year (95% CI: −5.1–1.7% per year).

Age cohorts were grouped by two-decade intervals starting at age 15 (15–34; 35–54; 55 and above). The rate of new cases declined notably by age: 7.3% per year (95% CI: 1.5–21.0% per year) in those aged 15–34; 1.6% per year (95% CI: 0.2–5.8% per year) in those aged 35–54; and 0 (97.5% CI: 0.0–5.0% per year) in those over age 55.

Aboriginal participants had a combined re-infection/incidence rate of 2.5% (95%CI: 0.8–5.9% per year), while all non-Aboriginal participants remained free from infection during the study period. Of 41 self-identified Inuvialuit participants, the rate of new cases was 3.3% per year (95% CI: 0.9–8.4% per year); among 24 self-identified Gwich'in participants the rate of new cases was 1.5% per year (95% CI: 0.4–8.2% per year). The estimated incidence rate difference comparing Gwich'in to Inuvialuit is −1.8% per year (95% CI: −6.1–2.5% per year); the wide CI indicates that this study is too small to accurately estimate the difference between these groups in the rate of new cases. The 2 participants who identified as “other Aboriginal” remained free from infection during the study period. Four out of 79 participants chose to not report their ethnic status.

## Discussion

Among participants in the AHPP aged 15 years and above, we observed an incidence proportion of 6.3% over an average follow-up of 3 years. In this population, the rate of new cases of *H. pylori* infection declined notably with age and was similar in males and females. Of note, all participants in this study aged 55 years and above remained free from infection throughout the study period, as did all non-Aboriginal participants. Due to the low incidence of new cases, we do not have enough data in this population sample to precisely estimate differences between ethnic and age groups. More data are needed from additional Canadian Arctic communities so that we can increase the size of regional population samples.

In this study, we have shown evidence suggesting that most AHPP participants treated for *H. pylori* infection remained free of infection during the follow-up period. Thus, we can infer that there was a corresponding decrease in the prevalence of *H. pylori* infection, bringing the prevalence in Aklavik closer to the average seen in southern Canada. This suggests a potential for reducing the burden of infection in communities like Aklavik through the application of screen and treat programs with long-term follow up. Before recommending such a strategy at the community level, however, a full assessment of costs and benefits is needed. Widespread antibiotic treatment may pose a risk to populations by increasing antibiotic resistance among *H. pylori* strains as well as other bacteria in the community. Our choice to screen and offer treatment to all positive participants is the result of consultation and consensus-building with the AHC, and it is appropriate in this community for a number of reasons. The AHPP has documented that *H. pylori* infection is highly prevalent in Aklavik, as is severe chronic gastritis and precancerous conditions of the stomach. The pattern observed in Aklavik fits with the observation that men in regional centres of the Northwest Territories have three times the rate of gastric cancer compared to men across Canada (15). The AHPP is part of a research program that is gathering similar data from other Canadian Arctic communities. In addition to assessing the burden of disease from *H. pylori* infection in this region, we will assess the cost-effectiveness of regional health care practices, which currently include testing for and treatment of *H. pylori* in patients presenting with a wide array of gastric symptoms that can be caused by factors other than *H. pylori* infection. The adoption of community health strategies aimed at reducing health risks from *H. pylori* infection requires careful generation and analysis of the full array of evidence that must be considered for effective public policy. An international analysis of reported re-infection rates estimated summary rates of 2.7% for developed countries (3,014 patients followed for 24–60 months) and 13% for developing countries (2,071 patients followed for 12–60 months) (16,17), but there is extensive variation within these groupings depending on the population studies. One study, for example, estimated the re-infection rate at 1.8% per year in a population of Brazilian patients with dyspeptic diseases (18). The AHPP is one of a very few sources of data on *H. pylori* incidence and re-infection rates in the circumpolar north. Our literature search identified just one report from this region. A 2-year prospective study of urban Alaska Native gastroenterology patients aged 18 years or above yielded an estimated re-infection proportion following successful treatment for *H. pylori* infection of 5.1% at 4 months, increasing to 7.2% at 6 months, 10.3% at 1 year and 14.6% at 2 years (19). Among Aklavik project participants who were treated for *H. pylori* infection, the estimated re-infection proportion was considerably lower at 4.7% over an average of 2.6 years at risk. Some of this difference may be attributable to differences between gastroenterology patients and a community-based study population as well as random variation; the Aklavik study population was younger on average than the Alaskan study population, so if *H. pylori* incidence decreases with age, differences in age distribution would not explain the observed difference re-infection frequencies. While *H. pylori* infection is generally more prevalent throughout North American Arctic communities for which we have data, compared to southern Canada and the United States, these findings suggest variation across Arctic communities, and demonstrate the need for data from additional communities to add to our knowledge of *H. pylori* infection and its related human health outcomes in the North.

## Conclusions

This incidence and re-infection study indicates that the screening and treatment components of the AHPP have substantially reduced the prevalence of *H. pylori* infection among project participants since the treatment component began in 2008. The CAN*Help* Working Group is continuing to collect data on determinants of treatment success as well as benefits of treatment in order to achieve our collaborative team's goals of addressing community concerns about health risks from *H. pylori* infection, and ultimately reducing these risks. Participant follow-up in Aklavik combined with epidemiological and ethnographic assessments of associated risk factors, and on-going community engagement and knowledge translation activities, are continuing in order to assess the long-term implications of community-wide test-and-treat programs for controlling *H. pylori* infection.
